# Evaluation of the Inhibitory Effects of Genipin on the Fluoxetine-Induced Invasive and Metastatic Model in Human HepG2 Cells

**DOI:** 10.3390/molecules23123327

**Published:** 2018-12-14

**Authors:** Yu-Syuan Tian, Kuan-Chou Chen, Nor Diana Zulkefli, Rida S. Maner, Chiu-Lan Hsieh

**Affiliations:** 1 Department of Biology, Changhua University of Education, 1 Jin-De Road, Changhua 50007, Taiwan; sherry0445@gmail.com (Y.-S.T.); nordianaiel@gmail.com (N.D.Z.); rida.maner912@gmail.com (R.S.M.); 2 Department of Uroloy, School of Medicine, College of Medicine, Taipei Medical University, 250 Wu-Xing Street, Taipei 11031, Taiwan; kuanchou@tmu.edu.tw; 3 Graduate Institute of Medicinal Sciences, College of Medicine, Taipei Medical University, 250 Wu-Xing Street, Taipei 11031, Taiwan

**Keywords:** hepatocellular carcinoma, fluoxetine, genipin, HepG2 cells, metastasis, invasion

## Abstract

Metastasis of hepatocellular carcinoma (HCC) is usually unrecognized before any pathological examination, resulting in time-taking treatment and poor prognosis. As a consequence, HCC patients usually show symptoms of depression. In order to suppress such psychiatric disorders and to facilitate better treatment outcome, antidepressants are prescribed. Up to present, information about the effect of antidepressants on HCC is still lacking. Therefore, we chose fluoxetine (FXT), one of the top five psychiatric prescriptions in the United States, together with the HepG2 cell model to explore its effect on HCC. Our study found that FXT (5 µM) increased the migratory distance of HepG2 cells by a factor of nearly 1.7 compared to control. In addition, our study also investigated the effect of genipin (GNP), which is an active compound from *Gardenia jasminoides* Ellis fruit (family *Rubiaceae*)*,* on the FXT-induced HepG2 cells. Our study found that 30 and 60 µM GNP reduced the migratory distance by 42% and 74% respectively, compared to FXT treatment alone. Furthermore, we also found that FXT upregulated matrix metalloproteinases (MMPs) genes, increased the protein expression of MMPs, urokinase-type plasminogen activator (uPA), nuclear factor kappa-light-chain-enhancer of activated B cells (NF-κB), activator protein 1 (AP-1), phosphorylated mitogen-activated protein kinase (P-p38), phosphorylated protein kinase B (P-Akt), downregulated tissue inhibitor metalloproteinases (TIMPs) genes and decreased the TIMPs proteins expression whereas, GNP fully counteracted the action of FXT. Conclusively, this study has provided valuable information regarding the possible molecular mechanisms through which FXT affects the metastatic invasiveness of HepG2 cells and evidences to support that GNP counteracts such effect via the same molecular mechanisms.

## 1. Introduction

Liver cancer is the sixth most common cancer in the world [1]. Cancer patients experience the symptoms of cancer and undergo cancer-related treatment, and may face the threat of recurrence and death in the future, resulting in a higher proportion of depression in cancer patients than average people. It is known that depression, fatigue and sleep disorders are inextricably linked in cancer patients undergoing treatment [2]. The incidence of depression in patients with liver cancer is higher than other people who suffer with general, chronic or other cancers; 51% of cancer patients experience sorrow and fatigue, 26% suffer from fatigue and sleep disorders and 50% experience sleep disorders and depression [3]. High incidence of severe depression is found to be extremely relevant to patient survival as severe depression can also make the immune system weaker and hence hinder survival. This may result in a condition where the treatment of liver cancer has effect on the patients’ both physical and psychological health. In order to prevent patients from this effects, the cancer treatment is usually supplemented with anti-depressant drugs to improve the sleep quality and stabilize the mood of the patients [4].

FXT, a type of selective serotonin reuptake inhibitor (SSRI), is an anti-depressant which is used to improve the psychological state of the patients [5]. However, it has certain potential side effects such as stress, insomnia, headache, anxiety and lethargy. Intake of FXT may cause spontaneous bleeding, lung injury and cardiotoxicity [6,7,8]. SSRI can inhibit the resorption of serotonin, thus increasing the amount of serotonin in the synapses and also indirectly increasing the amount of serotonin in the blood [9]. It was mentioned by Soll et al. [10], that serotonin triggers liver tumor proliferation in mice. A study pointed out that FXT increased the risk of breast cancer cells transferring to the brain in mouse model [11]. In the past, it has also found that FXT (less than 25 µM) promoted fat accumulation in primary mouse hepatocytes and easily formed fatty liver, which can cause liver hardening and liver cancer [12]. As per a study done by Stepulak et al. [13], FXT could ameliorate the inhibition of lung and colon cancer cells’ growth. It is still unclear whether FXT contributes to the proliferation or inhibition of different cancer cells, as the results from different studies vary. Also, there are no studies on the effect of FXT on the invasion and metastasis of liver cancer cells. Therefore, the influence of SSRI on the invasion and metastasis of liver cancer cells is worthy of further investigation.

Modern people’s health awareness is on the rise and being concerned of the side effects of conventional drugs. Therefore, in the medical treatment, the adjuvant therapy of natural and traditional Chinese medicine is increasingly promoted. The main active ingredient of the study is genipin (GNP) which is an active compound from *G. jasminoides*, a traditional Chinese medicine [14]. *G. jasminoides* exhibits certain pharmacological effects which are beneficial against inflammation, cancer, diabetes, angiogenesis, arthritis, etc. [15]. It has been shown that GNP induces antioxidation, anti-inflammatory, anti-ischemic, anti-hypertension effects on the rat models [16]. In anti-tumor studies, GNP could induce apoptosis in cervical cancer HeLa cells, liver cancer Hep3B cells, and prostate cancer PC3 cells, and showed that it ameliorated the inhibition of tumors and hyperplasia [17,18,19]. Another study showed that GNP can inhibit the invasion of liver cancer cells into normal liver tissues of mice [20]. Besides, it has also shown anti-depressant-like effects in mice by regulating monoamines and brain neurotrophic factors in the brain [21,22]. Therefore, in our study, we stimulated the human HepG2 cell line by FXT to investigate its effect on invasion and metastasis of HCC cells. Furthermore, we observed the rescue effect of GNP on the HepG2 cells after treatment with FXT.

## 2. Results

### 2.1. Effect of FXT-GNP Co-Treatment on the Cell Viability of HepG2 Cells

**Figure 1:**
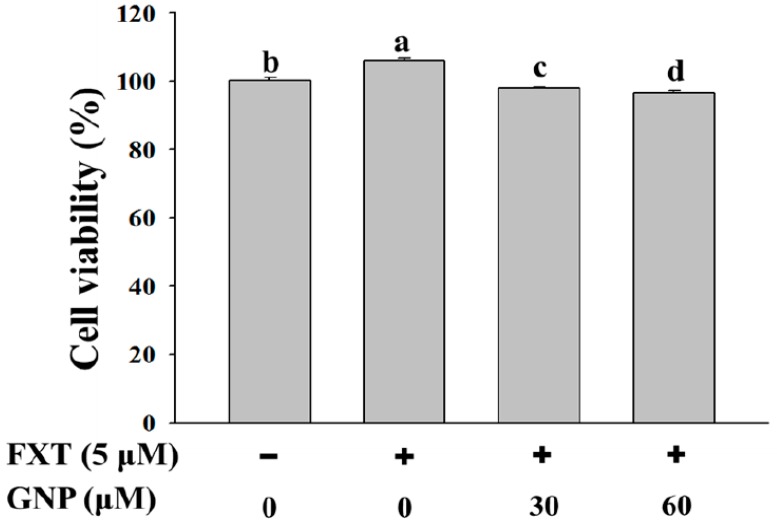
Effect of FXT-GNP on the cell viability of HepG2 cells. HepG2 cells were seeded onto a 6-well plate at 5 × 10^5^ cells/mL and incubated overnight, then treated with FXT-GNP at dose as indicated and further incubated for 72 h. DMSO (0.1%) was used as the vehicle control. Values are expressed as mean ± SD (*n* = 3). Different letters indicate statistical significance (*p* < 0.05).

The cell viability test of HepG2 cells was done in order to study the effect of FXT-GNP co-treatment on cell proliferation. HepG2 cells were seeded onto a 6-well plate at 5 × 10^5^ cells/mL and incubated overnight, then treated with FXT-GNP as the indicated dosage and further incubated for 72 h. Control was the HepG2 cells without FXT and GNP. Based on the Figure 1, the addition of 5 μM of FXT increased the cell viability by about 5% compared to the control (*p* < 0.05). Then with the addition of 30 and 60 μM of GNP co-treatment with 5 μM FXT, the cell viability decreased by about 7% and 9% respectively, compared with FXT treatment alone.

### 2.2. Migration Test on The HepG2 Cells Treated with FXT-GNP Co-Treatment

**Figure 2:**
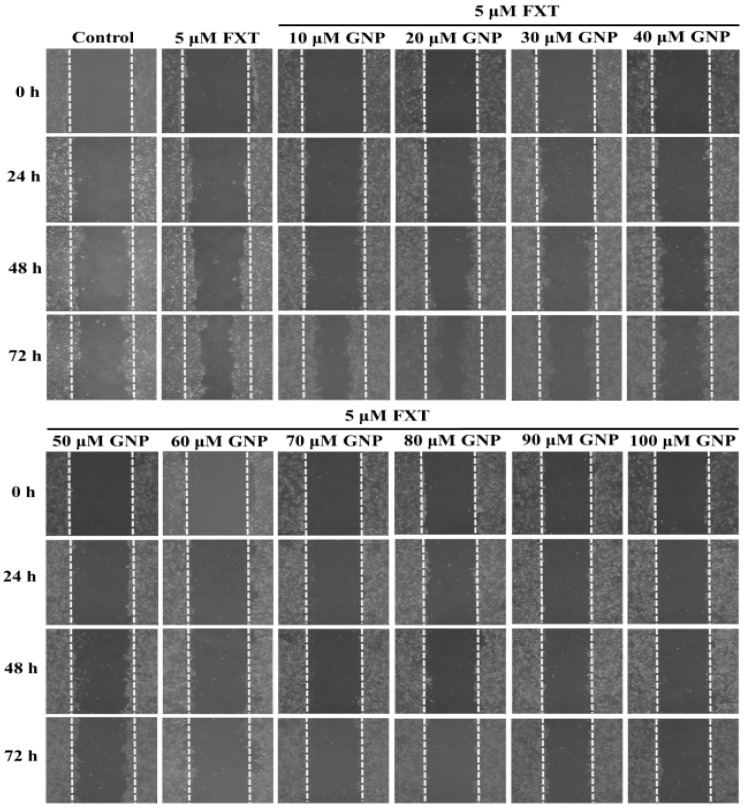

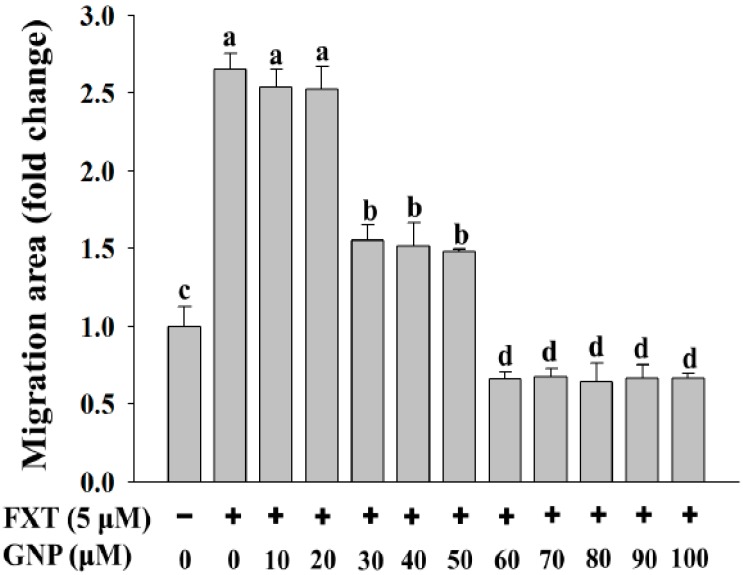
Migration test on the HepG2 cells treated with FXT-GNP. HepG2 cells were seeded onto a 6-well plate at 5 × 10^5^ cells/mL and incubated overnight, treated with FXT and varying dose of GNP as indicated and further incubated for 72 h. DMSO (0.1%) was used as vehicle control. Values are expressed as mean ± SD (*n* = 3). Different letters indicate statistical significance (*p* < 0.05).

The migration test was done on the HepG2 cells (Figure 2). The dotted lines (left and right) represent the area where the cells were attached (0 h). After 72 h of incubation, the cells started to migrate out from the attached area. Control was HepG2 cells without any application of FXT or GNP. FXT (5 μM) was added and results showed that it increased the migration area by about 1.7-fold compared to control (*p* < 0.05).

Then, with the co-treatment of 10 and 20 μM GNP, the migration area decreased but the differences were not significant compared to application of FXT alone (*p* > 0.05). However, the migration area decreased significantly with increased GNP concentration. It showed that, 30 μM of GNP-FXT (5 μM) co-treatment, decreased the migration area by about 42% compared to the FXT treatment alone (*p* < 0.05).

The addition of 40 and 50 μM GNP did not have significant difference from application of 30 μM GNP (*p* > 0.05). Then, with treatment of 60 μM GNP, the migration area of HepG2 cells decreased by about 74% compared to the FXT treatment alone (*p* < 0.05). The application of GNP at other concentrations such as 70, 80, 90 and 100 μM did not have that much significant difference with 60 μM GNP (*p* > 0.05). These results help us to infer that the application of GNP can decrease the cell migration caused by FXT, with increased concentration of GNP.

### 2.3. Matrigel Invasion Assay of FXT-GNP Co-Treatment on HepG2 Cells

**Figure 3:**
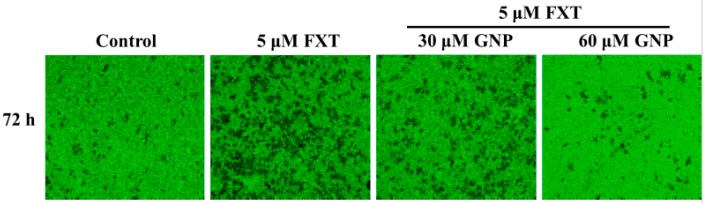

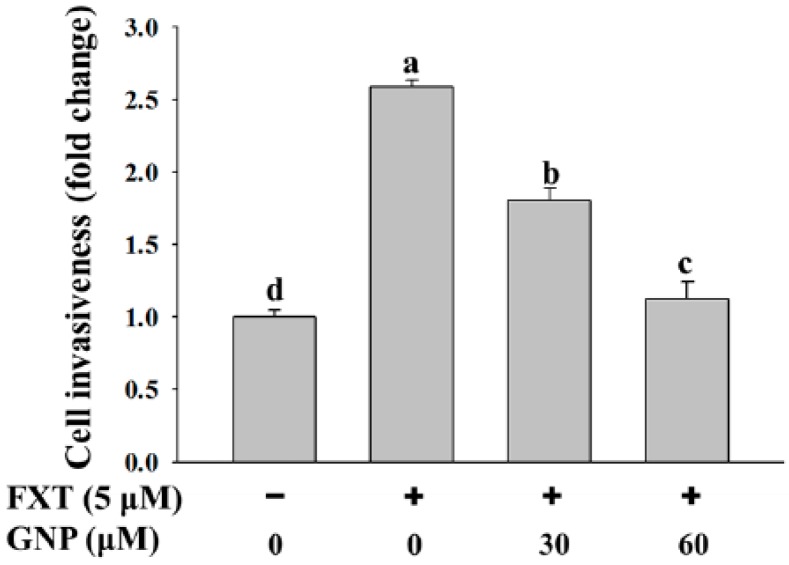
Matrigel invasion assay of FXT on HepG2 cells and the inhibitory effect of GNP. HepG2 cells were seeded onto a 24-transwell with serum-free medium at 5 × 10^4^ cells/mL and incubated overnight, then treated with FXT-GNP at dose as indicated and further incubated for 72 h. DMSO (0.1%) was used as the vehicle control. Values are expressed as mean ± SD (*n* = 3). Different letters indicate statistical significance (*p* < 0.05).

The study was performed with 30 and 60 μM GNP, as there were no significant differences at other concentrations (Figure 3). Based on the figure, the cell invasiveness increased significantly with application of FXT (5 μM) by about 1.6-fold compared to control (*p* < 0.05). However, with the co-treatment of 30 and 60 μM GNP, the cell invasiveness decreased by 31% and 54% respectively, compared to FXT treatment alone. This showed that higher concentration of GNP gave higher significant effects on the cell invasiveness (*p* < 0.05).

### 2.4. RT-PCR Analysis of Marker Gene Expression in HepG2 Cells After FXT-GNP Co-Treatment

**Figure 4:**
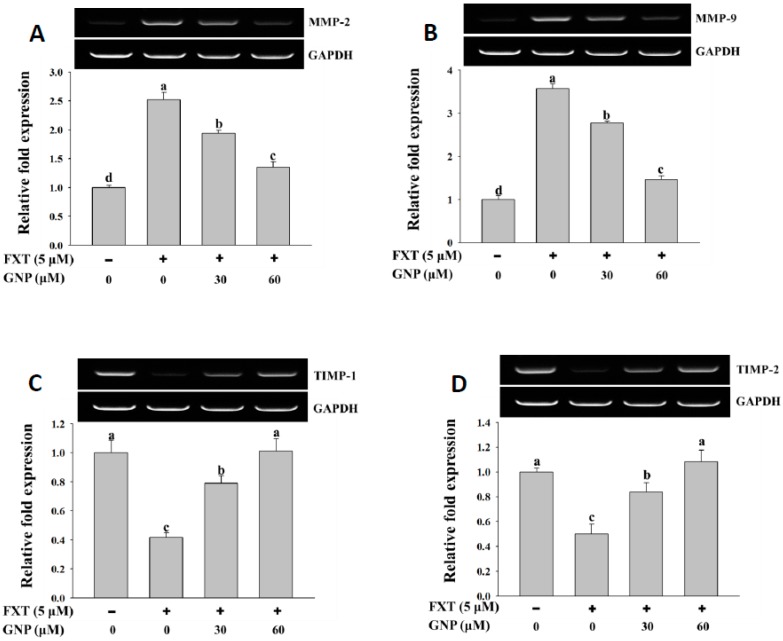
RT-PCR analysis of MMP-2 (**A**), MMP-9 (**B**), TIMP-1 (**C**) and TIMP-2 (**D**) gene expression in HepG2 cells after treatment with FXT and the attenuating effect of GNP. HepG2 cells were seeded onto a 6-well plate and incubated overnight, treated with FXT and GNP at dose as indicated and further incubated for 72 h. DMSO (1%) was used as vehicle control. Values are expressed as mean ± SD (*n* = 3). Different letters indicate statistical significance (*p* < 0.05).

The results of the RT-PCR analysis on the MMP-2 and MMP-9 gene expressions in HepG2 cells after treatment with FXT were shown in Figure 4A,B, respectively.

The application of 5 μM FXT increased the relative expression of the MMP-2 and MMP-9 significantly by about 1.5 and 2.6-fold, respectively, compared to control (*p* < 0.05). However, the co-treatment of 30 and 60 μM GNP- 5 μM FXT decreased the relative expression of MMP-2 by about 28% and 48% respectively, compared to FXT treatment alone. A similar trend was observed in MMP-9, where the relative expression decreased by about 22% and 58%, respectively (*p* < 0.05).

The results of RT-PCR analysis on TIMP-1 and TIMP-2 gene expression are shown in Figure 4C,D. The application of 5 μM FXT on the HepG2 cell decreased the relative expression of TIMP-1 and TIMP-2 by about 60% and 50%, respectively, compared to control (*p* < 0.05). The co-treatment of 30 and 60 μM GNP- 5 μM FXT increased the TIMP-1 expression significantly, by about 1.0 and 1.5-fold, respectively, compared to the FXT treatment alone. The trend was similar in TIMP-2, where the relative expression increased by about 68% and 1.2-fold, respectively (*p* < 0.05).

### 2.5. Western Blot Analysis of Protein Expression of Marker Gene in HepG2 Cells After FXT-GNP Co-Treatment

**Figure 5:**
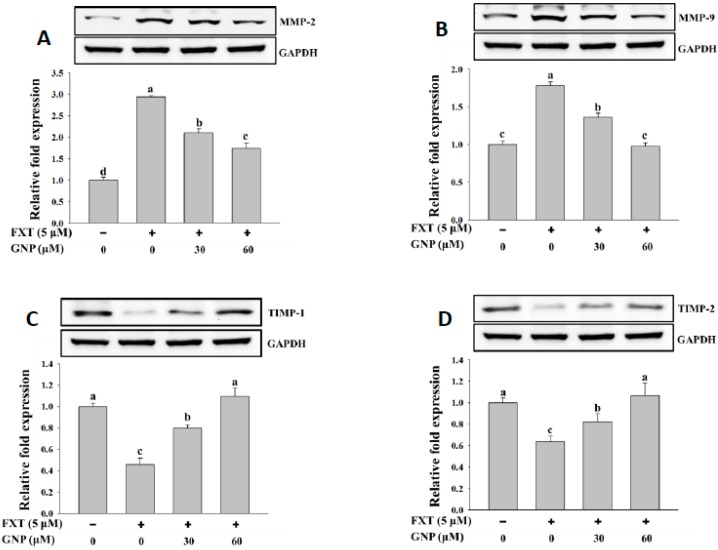

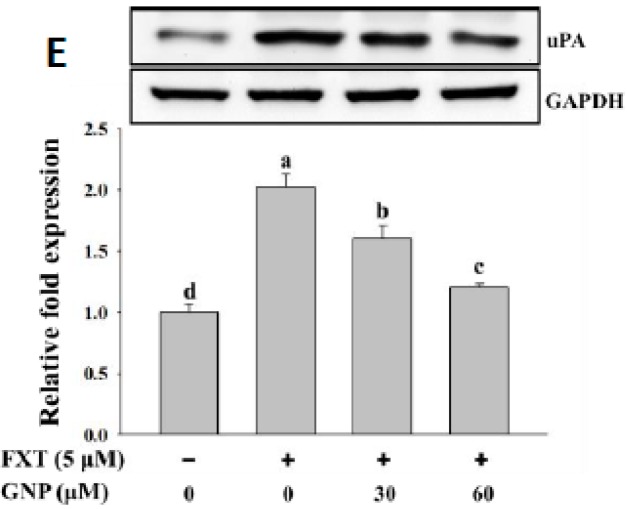
Western blot analysis of MMP-2 (**A**), MMP-9 (**B**), TIMP-1 (**C**), TIMP-2 (**D**) and uPA (**E**) proteins expression in HepG2 cells after treated with FXT and the attenuating effect of GNP. HepG2 cells were seeded onto a 6-well plate and incubated overnight, treated with FXT and GNP at dose as indicated and further incubated for 72 h. DMSO (0.1%) was used as vehicle control. Values are expressed as mean ± SD (*n* = 3). Different letters indicate statistical significance (*p* < 0.05).

The western blotting results of MMP-2 and MMP-9 are shown in Figure 5A,B, respectively. The addition of 5 μM FXT increased the expression of MMP-2 and MMP-9 protein significantly by about 2.0-fold and 75%, respectively, compared with control. The co-treatment of 30 and 60 μM GNP- 5 μM FXT decreased the MMP-2 protein expression by about 30% and 42%, respectively, compared with the FXT treatment alone. A similar trend was observed for MMP-9 where the protein expression decreased by about 23% and 43%, respectively. The results for protein expression of TIMP-1 and TIMP-2 in HepG2 cells are shown in Figure 5C,D, respectively. The addition of 5 μM FXT decreased the protein expression of TIMP-1 and TIMP-2 by about 55% and 36%, respectively, compared to control. However, with the co-treatment of 30 and 60 GNP with 5 μM FXT, the TIMP-1 protein expression increased significantly by about 78% and 1.3-fold, respectively, compared to FXT treatment alone. A similar trend was observed for TIMP-2 protein expression where it increased about by 28% and 67%, respectively (*p* < 0.05).

The result on the uPA protein expression in HepG2 cells is shown in Figure 5E. The addition of 5 μM FXT increased the relative expression significantly by 1.0-fold, compared with the control. After the co-treatment of 30 and 60 μM GNP-5 μM FXT, the protein expression decreased by about 20% and 43%, respectively, compared with the FXT treatment alone (*p* < 0.05).

### 2.6. Ameliorating Effect of GNP on the FXT-Induced MMP-2 and MMP-9 Activities in HepG2 Cells

**Figure 6:**
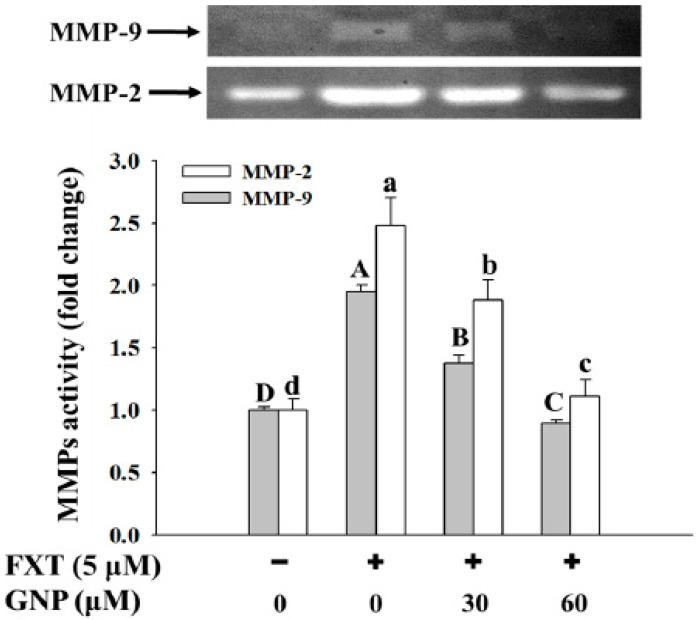
Ameliorating effect of GNP on the FXT-induced MMP-2 and MMP-9 activities in HepG2 cells. HepG2 cells were seeded onto a 6-well plate at 5 × 10^5^ cells/mL and incubated overnight, treated with FXT and GNP at dose as indicated and further incubated for 72 h. DMSO (0.1%) was used as vehicle control. Values are expressed as mean ± SD (*n* = 3). Different letters indicate statistical significance (*p* < 0.05).

The ameliorating effect of GNP on the FXT-induced MMP-2 and MMP-9 activities in the HepG2 cells is shown in Figure 6.

The activities of both the MMP-2 and MMP-9 increased significantly with the addition of the 5 μM FXT by about 1.5 and 1.0-fold respectively, compared with the control (*p* < 0.05). However, with the co-treatment of 30 and 60 μM GNP-5 μM FXT, the activity of MMP-2 decreased by about 28% and 56% respectively, compared with the FXT treatment alone. The similar trend was observed for MMP-9, where the activity decreased by about 30% and 60% respectively.

### 2.7. Western Blot Analysis of Signalling Pathway and Transcription Factors in HepG2 Cells after FXT-GNP Co-Treatment

**Figure 7:**
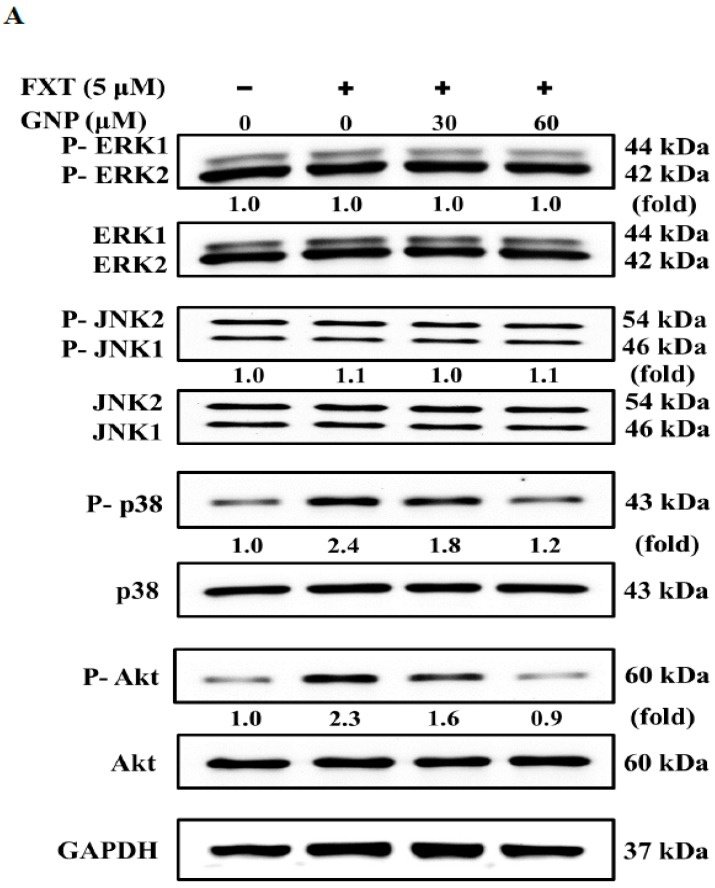

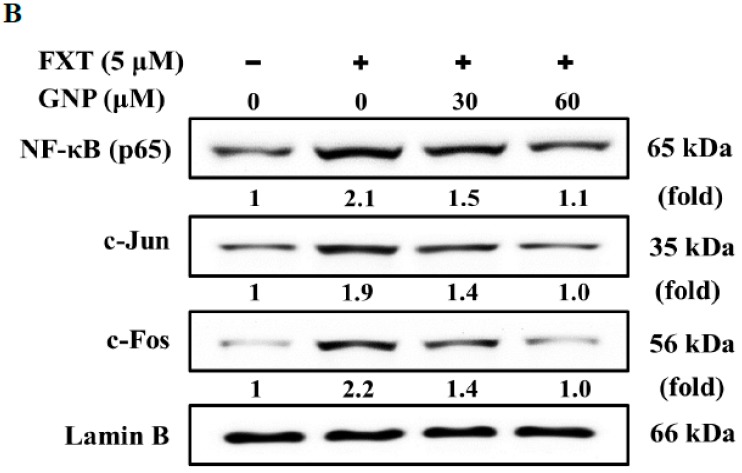
Western blot showing the expressions of p38 and Akt (**A**), transcription factors NF-κB and c-Jun/c-Fos (**B**) in HepG2 cells induced by FXT and the attenuating effect of GNP. HepG2 cells were seeded onto a 6-well plate and incubated overnight, then treated with FXT and GNP at dose as indicated and further incubated for 72 h. DMSO (0.1%) was used as vehicle control for 72 h. The intensity was subsequently quantified by the densitometer.

The results of the western blotting performed on cell signalling pathways are shown in Figure 7A. From the results, we observed that application of either FXT or FXT-GNP did not affect the phosphorylation of ERK1 and ERK2. The phosphorylation of JNK1 and JNK2 increased by about 10% when 5 μM FXT was applied, compared to control. When 30 μM GNP was added to FXT-induced cells, both proteins decreased, similar to the control. Next, the phosphorylated-p38 expression was increased by about 1.4-fold compared to control when 5 μM FXT applied. However, when 30 and 60 μM GNP were added to the FXT-induced cells, the p-p38 expression decreased by about 25% and 50% respectively, compared to FXT treatment alone.

For the Akt pathway, the p-Akt increased by about 1.30-fold with application of 5 μM FXT compared to the control. After the addition of 30 and 60 μM GNP, the protein expression decreased by about 30% and 61% respectively, compared to FXT treatment alone. The results of western blotting on the expression of transcription factors NF-κB and c-Jun/c-Fos are shown in Figure 7B. The application of 5 μM FXT increased the NF-κB expression by about 1.1-fold, compared with control. However, the co-treatment of 30 and 60 μM GNP-5 μM FXT decreased the expression by about 29% and 48%, respectively, compared with FXT treatment alone.

The expression of c-Jun was increased with application of 5 μM FXT by about 90%, compared with control. With application of 30 and 60 μM GNP, the expression decreased by about 26% and 47%, respectively, compared to the FXT treatment alone. For c-Fos, the expression increased by about 1.2-fold with application of 5 μM FXT. However, the expression decreased by about 36% and 55% with co-treatment of 30 and 60 μM GNP-5 μM FXT, respectively, compared with FXT treatment alone (*p* < 0.05).

## 3. Discussion

Metastatic invasion occurs when cancer cells proliferate up to a tumor diameter of more than 0.5 mm and the tumor cells can no longer obtain sufficient nutrients by diffusing in the surrounding cells and environment. Subsequently, the tumor cells release vascular endothelial growth factors (VEGF) to promote the proliferation of new blood vessels around the tumor cells and to supply nutrients through the blood to maintain the survival of cancer cells [23]. Angiogenesis not only provides adequate nutrition for cancer cells, but also provides another conduit for cancer cells to spread to other tissues or organs. In the present study, we demonstrated that FXT could significantly enhance the HCC cells migration and invasion in vitro. This may be due to the ability of serotonin to activate angiogenesis in human endothelial cells through angiogenic processes similar to VEGF, such as G-protein-coupled receptors to activate Src, phosphorylation of PI3K-AKT-mTOR and p70S6K signalling proteins which in turn promote angiogenesis [24].

The MMP-2 and MMP-9 are one of the types of matrix metalloproteinases, which are mainly composed of type IV collagen, a major component of the basement membrane. According to the literature, high metastatic lung cancer, rectal cancer and breast cancer are found to have high MMP-2 and MMP-9. Therefore, the activity or amount of MMP-2 and MMP-9 in tumor tissues is often used as tumor indicator in terms of activity and severity [25,26,27]. The regulation of MMPs activity is mainly through three different pathways: transcription, inhibition of protein expression and activation of proteinase. Through this study, we found that 5 μM of FXT significantly increased the expression of MMP-2 and MMP-9 mRNA in HepG2 cells while the results were reversed with the co-treatment of FXT with GNP.

The main signalling pathways for the regulation of MMPs are the mitogen-activated protein kinases family (MAPK) and phosphatidylinositol 3-kinase (P13K)/protein kinase B (Akt) pathways, while the activation of transcription factors AP-1 and NF-κB increased the expression of MMPs [28,29,30]. Inferred from the results of the signalling pathway and transcription factors, FXT may activate NF-κB (p65) into the nucleus via NF-κB binding site by increasing phosphorylation of Akt signalling protein. The c-Jun and c-Fos proteins are members of the AP-1 transcription factor family which involved in the regulation of gene expression during the cell cycle [31]. FXT may also increase the expression of AP-1 family members, c-Jun and c-Fos proteins, by increasing the phosphorylation of p38/MAPK message protein and enter the nucleus to bind to the AP-1 binding site, thereby inducing the gene expression of MMP-2 and MMP-9 in HepG2 cells. However, with the application of GNP on the FXT-induced HepG2 cells may reduce the expression of NF-κB (p65) and AP-1 protein in NF-κB and AP-1 binding sites in the nucleus by inhibiting phosphorylation of Akt and p38/MAPK signalling proteins in order to achieve inhibition of MMP-2 and MMP-9 gene expression. In addition, both FXT and GNP had no effect on extracellular signal-regulated kinases 1/2 (ERK1/2) and stress-activated protein kinases (SAPK)/Jun amino-terminal kinases (JNK) signal protein phosphorylation.

**Figure 8:**
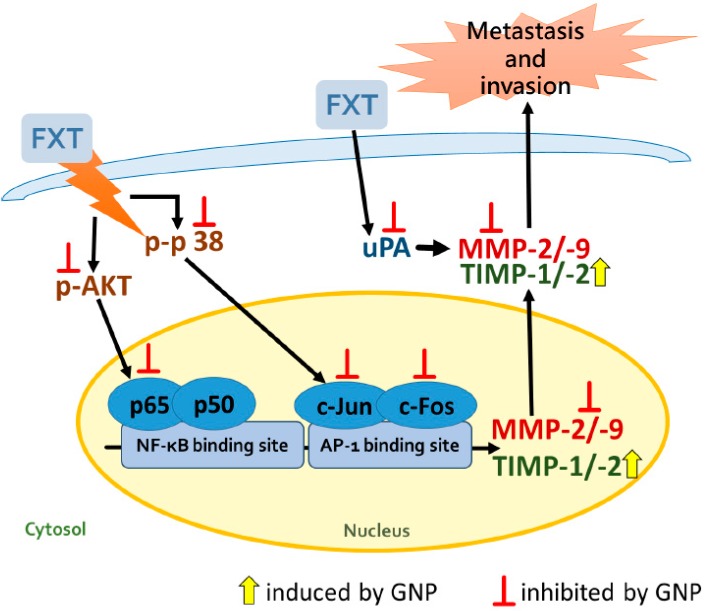
Proposed signal transduction pathways of GNP to inhibit the FXT-induced cell metastasis and invasion of HepG2 cells.

The activity of translated MMPs is affected by TIMPs and uPA. When cell MMPs are overreactive, in order to avoid cell damage, TIMPs can specifically block the activity of MMPs by binding to the Zn-binding site of activated MMPs. On the other hand, uPA is a proteolytic enzyme, which can activate plasminogen into plasmin, cleaves MMPs by plasmin, and then activates MMPs. The interactions between MMPs, TIMPs and uPA stabilize the reaction of MMPs [32,33]. From the results, it was found that 5 μM of FXT reduced the expression of TIMP-1 and TIMP-2 mRNA in the transcriptional stage, which reduced the performance of both proteins, thereby increased the MMP-2 and MMP-9 expression. The effect of GNP on the expression of TIMP-1 and TIMP-2 in FXT-induced HepG2 cells was to increase the expression of TIMP-1 and TIMP-2 mRNA through transcriptional stage. In turn, the inhibitory effect on MMP-2 and MMP-9 increased, and thus the activity of MMPs decreased. Our results were consistent with other studies, where GNP has shown to enhance the protein expression of TIMPs by the signal pathway of p38 MAPK, which inhibits MMP protein and the invasion and metastasis of human hepatoma HepG2 and MHCC97L cells [20]. The result showed that 5 μM of FXT increased the expression of uPA protein, which in turn increased the MMPs after translation, and promoted the activation of MMP-2 and MMP-9. Other studies also reported that FXT significantly increased uPA in human embryo [34]. The expression of uPA protein in HepG2 cells decreased after GNP was applied. In turn, it affected the translation of MMPs and reduced the activation of MMP-2 and MMP-9. The proposed signal transduction pathway of GNP to inhibit the FXT-induced cell metastasis and invasion of HepG2 cells is shown in Figure 8. These findings suggested that GNP can be a potential inhibitor for metastasis and invasion and thus could be considered as an adjuvant which reduces the side effects of FXT in liver cancer patients.

## 4. Materials and Methods

Tissue culture media, fetal bovine serum (FBS) and supplements were purchased from Gibco (Rockville, MD, USA). GPSAPDH was purchased from Cusabio (Wuhan, China). GNP, and FXT were purchased from Sigma (St. Louis, MO, USA). Anti-MMPs, anti-TIMPs, anti-uPA and uPA were purchased from Protein Tech (Chicago, IL, USA). Anti-ERK, anti-P-ERK, anti-JNK, anti-P-JNK, anti-p38, anti-P-p38, anti-AKT, and anti-P-AKT were purchased from Cell Signaling Technology (Danvers, MA, USA). Anti- NF-κB, anti-c-Jun, and anti-c-Fos were purchased from Merck Milipore (Burlington, MA, USA). HRP-conjugated secondary antibodies were purchased from Bio-Rad (Madrid, Spain).

### 4.1. The Culture and Subculture of Cells

HepG2 liver cancer cells from a frozen tube were quickly thawed in a 37 °C water bath and inoculated into DMEM medium. The culture was cultured in an incubator with constant temperature of 37 °C, containing 5% of CO_2_ and the culture medium was changed every two to three days until the growth of cells reached confluence of 90%. The cells were digested with 0.05% trypsin-EDTA (TE), and culture medium was added to stop the reaction. The ratio of cell to the fresh medium was at 1:2 or 1:3. When the adherent cells were already about 80% confluent, cell subculture can be done and cryopreserved at −80 °C for storage.

### 4.2. Cell Viability MTT Test

HepG2 liver cancer cells were cultured in 6 well plate (5 × 10^5^ cells/mL), respectively and incubated few days (4–6 days) to let differentiation happen. Then, different concentrations of drugs were added and after the cell dosing reaction time was terminated, the old cell culture solution was removed and the cells were washed twice with PBS solution to remove the culture solution. Appropriate amount of PBS solution and 10% MTT solution were added and incubated for 30 min to 3 h. Removed the MTT solution, and added 0.5 mL of DMSO and allowed to stand in the dark room for 2 h. The DMSO solution was sucked out and ELISA Reader (Thermo Fisher Scientific Inc., Waltham, MA, USA) was used to measure the absorbance at 570 nm. Cell viability was calculated using the following formula: Cell viability (%) = [Abs 570 nm (sample)/Abs 570 nm (control)] × 100%.

### 4.3. Cell Migration Assay

The wound healing ability of HepG2 liver cancer cells was observed using cell migration assay. HepG2 liver cancer cells were pre-cultured in 6 well plate (5 × 10^5^ cell/mL), and cultured until the cell density was about 90%. The culture solution was aspirated and a blank area was drawn in the middle of the well with a 1 mL micropipette tip. The smeared cells were removed with PBS solution, and the PBS solution was aspirated, and the culture solution was added and dosing reaction was carried out. An inverted phase difference optical microscope (Culture Microscope Model CKX41, Olympus, Tokyo, Japan) was used at 0, 24, 48, and 72 h to observe and photograph the morphology of cell migration. The Image J software was used to quantify the cell migration area for statistical analysis.

### 4.4. Cell Invasion and Metastasis Assay

After cutting HepG2 cells with TE, 5 × 10^4^ cells/mL were seeded into the upper layer of each Matrigel (200–300 μg/mL)-coated transwells (pore size of 8 μm). After 12 h of placement, the upper layer of the porous membrane was replaced with serum-free DMEM and different concentrations of FXT were added. 10% fresh FBS culture solution was added to the lower layer with different concentrations of FXT and incubated in a 5% CO_2_ and 37 °C incubator. After the incubation time, the cells that were not transferred to the lower layer were carefully scraped with a cotton swab, fixed with methanol for 10 min and stained with crystal violet (0·08 g/mL EtOH) for 1 h. A vertical phase difference optical microscope (Culture Microscope Model CKX41) was used to photograph the morphology of cell invasion and metastasis. Image J software was used for statistical analysis.

### 4.5. Analysis of Gene Expression in HepG2 Liver Cancer Cells

#### 4.5.1. RNA Extraction and Drug Reaction Termination of Cell Samples

TRIzol kit was used to extract the RNA from cells. The cells were first washed twice with a sterile PBS solution, 1 mL/well Tri-Reagent (Sigma, Poole, England) was added, and the cells were repeatedly mixed with a pipette several times until the solution was no longer viscous, and the solution was placed in a sterile Eppendorf. The reaction was continued at room temperature for 3 min. Chloroform (0.2 mL) was added and shaken vigorously for 15 s, allowed to stand at room temperature for 5 min, and then centrifuged at 12,000 rpm for 15 min at 4 °C. The topmost RNA was carefully pipetted into another new Eppendorf and isopropanol (0.5 mL) was added, gently mixed up and down 10 to 15 times, allowed to stand for 5 min and then centrifuged at 12,000 rpm for 15 min at 4 °C. The supernatant was carefully poured off and the resulting white precipitate was the RNA extract, which was rinsed with 1 mL of 75% alcohol per 1 mL of Tri Reagent, and the extract centrifuged at 7500 rpm and 4 °C for 5 min. The supernatant was removed and the washing repeated twice. The dried RNA extract was added to 50 μL of diethyl pyrocarbonate (DEPC)-treated water and the Nano-Drop 1000 spectrophotometer was used to determine the extraction effect and the concentration by measuring the OD_260/280_ ratio (A_260_/A_280_ = 1.7–1.9). The RNA was stored at −80 °C.

#### 4.5.2. Reverse Transcription Polymerase Chain Reaction (RT-PCR)

The same concentration of RNA obtained from the Nano-Drop 1000 spectrophotometer was used for reverse transcription reaction in preparation of cDNA. The reagents were as follows: 2 μL RNA (1 μg~10 pg), 10 μL 2× Fast premix, 2 μL Primer mix, 1 μL 25% DMSO, and 4 μL ddH_2_O. The total volume would be 19 μL. The mixture is placed in a dry bath at 65 °C for 5 min, and immediately placed on ice for 1 min, then reverse transcriptase (1 μL) was added. The total volume was 20 μL The mixture was incubated in the PCR machine at a temperature of 42 °C for 30 min, followed by 85 °C for 5 min, and stored at −20 °C till use.

#### 4.5.3. Polymerase Chain Reaction (PCR)

The expression of MMP 2/9 and TIMP 1/2 genes in HepG2 liver cancer cell lines were analyzed by PCR. The reagents used were as follows: 3 μL of cDNA, 2 μL of forward primer (10 μM), 2 μL of reverse primer (10 μM), 12.5 μL of 2× PCR Master Mix, 4.5 μL of ddH_2_O, and 1 μL of 25% DMSO. The total volume would be 25 μL. The PCR condition was as follows: the first cycle was pre-denaturation at 94 °C for 5 min, the next 35 cycles were for denaturation at 94 °C for 30 s, annealing (GPSAPDH; at 60 °C for 30 s, MMP 2/9 at 58 °C for 1 min, TIMP 1/2 at 58 °C for 30 s), and extension at 72 °C for 30 s. The next one cycle was final extension at 72 °C for 10 min. The final cycle was cooling down at 4 °C for 10 min. The sequences of the primer genes are given in Table 1.

#### 4.5.4. DNA Gel Electrophoresis

The concentration of the agar used in this paper was 1.7%. The appropriate amount of agar powder was weighed and mixed into 0.5× TAE buffer. The mixture was heated using a microwave, then 10 μL/100 mL of safe view DNA Stain was added allowed to cool down a bit at room temperature, about 10 min, and poured into the plastic cast tray. The gel was cooled avoiding the light until it solidified. 0.5× TAE was used as the electrophoresis buffer. The PCR products were injected into the agar and 100 Volt was applied for 30 min. Then, the images were captured using the Luminescence/UV Image System and the results were compared with the DNA ladder. The images were kept for quantitative analysis.

#### 4.5.5. Gelatin Zymography Analysis

The enzyme activity of MMP-2/9 was analyzed using gelatin zymography in cell supernatant. The main principle was to use sodium dodecyl sulfate-polyacrylamide gels electrophoresis (SDS-PAGE) containing proteinase matrix to detect protein enzyme activity. HepG2 cells were seeded in a 6 well plate, cultured in DMEM medium and intervened in the FXT. After 48 h of culture, the culture solution was removed, and then cultured in serum-free DMEM medium and then intervened in the FXT, and the culture was continued for 24 h. The culture solution was taken as a sample. The sample and the 5× non-reducing protein loading dye were mixed at a ratio of 4:1, then injected and run at 70 V. After 20 min, changed to 140 V and run for 50 min. After washing with renature buffer for 30 min, added 50 mL of reaction buffer and incubated at 37 °C (70 rpm) for 16 h, then stained with Commassie Blue for 30 min, then developed, photographed, and analyzed quantitatively.

### 4.6. Protein Signal Protein and Transcription Factor Protein in HepG2 Analysis

#### 4.6.1. Protein Extraction

After the dosing reaction of HepG2 cells in 6 well plate was terminated, the culture solution was removed. The cells were repeatedly washed twice using iced PBS, added about 300 μL of RIPA buffer to each well and placed on ice. After 5 min, carefully scraped the cells and collected them in an Eppendorf. Disrupted the cells using shaker and put on ice for 10–20 min for every 10 min, three times in total and then centrifuged at 13,000 rpm for 5 min at 4 °C. The supernatant was placed into new Eppendorf and stored in −80 °C for further use.

#### 4.6.2. Cytoplasmic and Nuclear Protein Preparation

Cytoplasmic and nuclear proteins were extracted with a Nuclear/Cytosol Fractionation Kit (BioVision, Milpitas, CA, USA). The procedure was maintained at 4 °C. After the dosing reaction of HepG2 hepatoma cells cultured on 6 well plates was terminated, the culture solution was removed and repeatedly washed twice with iced PBS, and then 200 μL of LCEB-A mix (Cytosol extraction buffer A, DTT, protease inhibitor) were added to each well. The cells were carefully scraped and placed in a new Eppendorf, mixed with a pipette and shaken at high speed for 15 s. After 10 min on ice, 11 μL cytosol extraction buffer B (CEB-B) was added to each tube, vortexed for 5 s at high speed, put on ice for 1 min, shaken for another 5 s, centrifuged at 13,000 rpm for 5 min at 4 °C, and the supernatant collected in a new Eppendorf (cytoplasmic protein liquid) placed on ice. Each tube of the precipitate (containing the nucleus) was added with 100 μL of nuclear extraction buffer (NEB) mix (DTT, protease inhibitor), and the precipitate was carefully dispersed by pipet. After high-speed shaking for 15 s, the tube was placed on the ice for 10 min and this step repeated four times. Then, after centrifugation at 13,000 rpm for 10 min at 4 °C, the supernatant was collected into a new Eppendorf (nuclear protein solution). The cytoplasm and nuclear protein solution was kept in a −80 °C freezer for later use.

#### 4.6.3. Protein Quantification

Protein was quantified using Bradford protein-binding assay method, based on the conjugation between the Comassie Brilliant Blue G-250 and the proteins. The color of G-250 and protein binding solution would change from red to blue and the absorbance value was detected at 570 nm wavelength. The albumin at a known concentration of 24 mg/mL was serially diluted to a concentration of 12, 6, 3, 1.5, 0.75 and 0 mg/mL. The protein assay was diluted to 1:4 and 998 μL of the protein extract was added to the 2 μL of standard solution and mixed uniformly. The absorbance was detected at a wavelength of 570 nm and a standard concentration curve was prepared. The protein concentration of the sample was determined by comparing the absorbance of the sample against the standard concentration curve.

#### 4.6.4. Polyacrylamide Gel Electrophoresis Analysis

The protein electrophoresis method uses SDS-PAGE to separate proteins according to different molecular weights. The gel-completed film was placed in an electrophoresis tank, and a running buffer was added. The protein sample was mixed with 5× protein loading dye, heated at 100 °C for 10 min. The samples were sequentially injected into the pores of the colloid and passed through a voltage of 70 V. After the sample was passed through the stacking gel, the voltage was adjusted to 140 V. Adjusted the electrophoresis time according to the need.

#### 4.6.5. Western Blotting Method

The electrophoresis film was immersed in the ddH_2_O for 5–10 min, then replaced with transfer buffer and vortexed for 10 min. The PVDF membrane was cut to meet the size of the electrophoresis film, activated with methanol for 5–10 min, and a full wet transfer device used, whereby the protein transfer was done for 2 h at 400 mA. Then, the membrane was removed and soaked in the blocking buffer at room temperature for 1 h and vortexed for 10 min and washed with a 1× TBST buffer for 10 min three times. The desired primary antibody was added and placed at 4 °C overnight. The next day, it was washed with 1× TBST buffer for 10 min three times, then the secondary antibody corresponding to the primary antibody was added, and the reaction was performed at room temperature for 2 h, and then washed with 1× TBST buffer for 10 min three times. Then, the PVDF membrane was immersed in chemiluminesence HRP substance (Millipore, Bedford, MA, USA) for 1 min, and performed imaging and analysis. The antibodies used for MAPK, SAPK/JNK, p38 MAPK and AKT were from Cell Signalling (Danvers, MA, USA). For uPA, MMP2, MMP9, TIMP1, TIMP2, and Lamin B were from Protein Tech (Chicago, IL, USA). GAPDH was from Cusabio (Wuhan, China).

### 4.7. Statistical Analysis

The statistical significance was analyzed using Statistical Package for the Social Sciences (SPSS) version 15.0 software (SPSS Inc., Chicago, IL, USA). The data were expressed as mean ± standard error (mean ± SD) and one-way analysis of variance (one-way ANOVA) was used for the difference analysis in each test group. The Duncan’s multiple range test was used to analyze the average between the test groups. A *p* value less than 0.05 (*p* < 0.05) was considered as statistically significant. Different letters in the results represent that there was significant difference and vice versa.

## 5. Conclusions

In our study, we found that FXT promoted the invasion and metastasis of human HepG2 cells and GNP ameliorated the adverse effect of FXT. Hence, this study shows that GNP can be a potent complementary and alternative medicine (CAM) and as an adjuvant in the cancer treatment, thus providing a basis for the further clinical research.

## Figures and Tables

**Table 1:** Primer sequences.

Gene	Primer	Size (bp)
MMP2	Forward 5′-TGGCAAGTACGGCTTCTGTC-3′ Reverse 5′-TGGCAAGTACGGCTTCTGTC-3′	180
MMP9	Forward 5′-CACTGTCCACCCCTCAGAGC-3′ Reverse 5′- GCCACTTGTCGGCGATAAGG-3′	263
TIMP1	Forward 5′-CAAGATGACCAAGATGTATAAAGG-3′ Reverse 5′-AACAGTGTAGGTCTTGGTGAAG-3′	247
TIMP2	Forward 5′-CAGCTTTGCTTTATCCGGGC-3′ Reverse 5′-ATGCTTAGCTGGCGTCACAT-3′	176
GAPDH	Forward 5′-ACCACAGTCCATGCCATCAC-3′ Reverse 5′-ACCACAGTCCATGCCATCAC-3	452
